# Supplementary material for: The genome sequence of the Tawny Oak Tortrix, Acleris ferrugana (Denis & Schiffermüller), 1775 (Lepidoptera: Tortricidae)
Source: Wellcome Open Res. 2025 Sep 15;10:507. [Version 1] doi: 10.12688/wellcomeopenres.24876.1 (PMC12605580; doi:10.12688/wellcomeopenres.24876.1)
Supplement: Peer review history

# Reviewer report: Reviewer response for version 1

**DOI:** 10.21956/wellcomeopenres.27400.r136447

**Published:** 2025-11-12

**Authors:** Noah H Rose

**Affiliations:** 1 University of California San Diego, San Diego, USA

This manuscript describes a newly assembled Acleris ferrugana (rusty oak button) genome. The combination of long read sequencing and Hi-C scaffolding is appropriate, the assembly methods are well suited to the data, and the resulting assembly appears to be of high quality. The species in question is common across the British and Irish Isles, so this assembly will be a valuable resource for the Darwin Tree of Life Project.

Are sufficient details of methods and materials provided to allow replication by others?

Yes

Is the rationale for creating the dataset(s) clearly described?

Yes

Are the datasets clearly presented in a useable and accessible format?

Yes

Are the protocols appropriate and is the work technically sound?

Yes

Reviewer Expertise:

Evolutionary genetics, ecological genomics

I confirm that I have read this submission and believe that I have an appropriate level of expertise to confirm that it is of an acceptable scientific standard.

# Reviewer report: Reviewer response for version 1

**DOI:** 10.21956/wellcomeopenres.27400.r136449

**Published:** 2025-11-11

**Authors:** Piotr Zieliński

**Affiliations:** 1 Jagiellonian University, Kraków, Poland

This genome note presents the first high-quality reference genome assembly for *Acleris ferrugana*, the Tawny Oak Tortrix, generated as part of the Darwin Tree of Life (DToL) project. The assembly was based on PacBio HiFi, Hi-C, and RNA-seq data from specimens collected in Wytham Woods, UK. The resulting genome totals 480.03 Mb, with 99.03% of the sequence scaffolded into 31 chromosomal pseudomolecules, including the W and Z sex chromosomes. The mitochondrial genome was also assembled (16.58 kb).

 The authors followed standard DToL protocols for sample collection, barcoding, sequencing, assembly, and curation. The genome achieved a high level of completeness and accuracy (BUSCO 98.2%). Overall, this article represents a technically sound and well-documented contribution to the growing catalogue of lepidopteran genomes. The data provide valuable resources for future population genomic research on Tawny Oak Tortrix, its evolution, ecology, and comparative genomics.

**Assembly completeness and haplotype representation**

 The authors report that the primary assembly represents 86.3% k-mer completeness. The relatively low completeness for the primary assembly might merit a short discussion — for example, whether it reflects biological reality or technical filtering?

**Chromosome assignment and sex determination**

 The manuscript states that the W chromosome remained unscaffolded due to the Hi-C data being derived from a male individual. While this limitation is understandable, the authors could briefly note how this affects completeness of the sex chromosome assembly and whether future sequencing of a female could improve this.

 Table 3 lists chromosome-level scaffolds, but sex chromosomes sizes appear very large. A brief comment on the possible reasons for that, would be useful.

**Overall Assessment**

 This manuscript represents a high-quality genome resource note, aligning with the standards of the Darwin Tree of Life initiative. The technical execution is excellent, and the data are fully open and reusable.

Are sufficient details of methods and materials provided to allow replication by others?

Yes

Is the rationale for creating the dataset(s) clearly described?

Partly

Are the datasets clearly presented in a useable and accessible format?

Yes

Are the protocols appropriate and is the work technically sound?

Yes

Reviewer Expertise:

Population genomics, molecular ecology

I confirm that I have read this submission and believe that I have an appropriate level of expertise to confirm that it is of an acceptable scientific standard.

# Reviewer report: Reviewer response for version 1

**DOI:** 10.21956/wellcomeopenres.27400.r136450

**Published:** 2025-11-10

**Authors:** Shangzhe Zhang

**Affiliations:** 1 University of St Andrews, St Andrews, UK

The authors present a high-quality genome assembly for *Acleris ferrugana*, providing a valuable genomic resource that contributes meaningfully to the Tree of Life initiative. The sequencing strategy, assembly pipeline, and overall analytical workflow are robust, and the results appear well supported. I recommend acceptance of this work after addressing a few minor but important points.

(1) Sample information and mapping statistics:

 Multiple samples were used across different sequencing platforms. This raises potential concerns regarding mapping consistency and sequence heterogeneity. It would be helpful to include a brief summary of the mapping statistics in the Results section to confirm data concordance. Additionally, more details about the samples should be provided—specifically, the sex of each individual and whether they were collected from the same wild population or derived from an inbred laboratory line.

(2) Identification of the Z chromosome:

 The method used to identify the Z chromosome is not clearly described and appears to be missing from the Methods section. Please provide additional information or specify the pipeline used for chromosome identification to enhance reproducibility.

(3) Chromosome number and karyotype consistency:

 Chromosome number is often an informative feature in Lepidoptera genomics. It would be valuable to comment on whether the assembly’s inferred chromosome count aligns with previously reported karyotype stability in related species.

(4) Clarification on the Z chromosome scaffolding:

 In the statement, “Most of the assembly sequence (99.03%) was assigned to 31 chromosomal-level scaffolds,...” it is unclear whether the Z chromosome was successfully scaffolded or identified. Please clarify this point.

Are sufficient details of methods and materials provided to allow replication by others?

Yes

Is the rationale for creating the dataset(s) clearly described?

Yes

Are the datasets clearly presented in a useable and accessible format?

Yes

Are the protocols appropriate and is the work technically sound?

Yes

Reviewer Expertise:

Genomics, Evolutionary biology

I confirm that I have read this submission and believe that I have an appropriate level of expertise to confirm that it is of an acceptable scientific standard, however I have significant reservations, as outlined above.

# Reviewer report: Reviewer response for version 1

**DOI:** 10.21956/wellcomeopenres.27400.r134540

**Published:** 2025-10-14

**Authors:** Luana Bataglia

**Affiliations:** 1 Catholic University of the Sacred Heart - Piacenza and Cremona Campus (Ringgold ID: 96985), Piacenza, Emilia-Romagna, Italy

The article reports the sequencing of the genome of the Lepidoptera species *Acleris ferrugana*. 

 1) Although the article type is a data note and focuses on technical results, the background requires more biological information about the species to justify the creation of the dataset.

 Some points to be considered are:

 -What is the biological/conservational importance of the species?

 -Is this species considered vulnerable?

 -Can it be considered an insect pest? 

 -The authors mentioned the *Acleris ferrugana *as a variable species. Therefore, should the sequencing be related to more phylogenetic or evolutionary aims? 

 The authors should clarify the interest in sequencing this species.

 2) The sex of the sequenced specimens should be specified in the methods section. The only specification present is for the genomic DNA (female). Only in the results section is it reported that a male was used for the HI-C sequencing. There is no information about the specimen used in RNA-seq.

 3) For better and faster understanding, the authors should report in the text which body parts were used for DNA barcoding and which remaining parts were used for sequencing. 

 4) The mitochondrial genome data were poorly reported. The only information is about the length of 16.58 kilobases, reported in the abstract. But this information is missing in the results section. 

 5) The same for the RNA-seq data, which needs to be further explored.

 6) Although it is written that the gene annotation is still to be done, I strongly suggest that the authors complete it as soon as possible and include this information in the article.

 7) The authors should include small discussions related to the resulting data to add a minimum degree of biological interpretation:

 Example: - Do the length of the nuclear and mitochondrial genomes match the expected size and resemble those of close species?

 -The same kind of discussion should be done with the annotation data. Do the number of predicted genes and proteins match the expected number and resemble those of close species?

 - Some groups of genes presented a significant increase or decrease in the species?

 - Did the authors detect potential unique regions/highlights in the genome of this species?

Are sufficient details of methods and materials provided to allow replication by others?

Partly

Is the rationale for creating the dataset(s) clearly described?

No

Are the datasets clearly presented in a useable and accessible format?

Partly

Are the protocols appropriate and is the work technically sound?

Yes

Reviewer Expertise:

Molecular Biology, Genetics, Epigenetics, Entomology, Biotechnology.

I confirm that I have read this submission and believe that I have an appropriate level of expertise to confirm that it is of an acceptable scientific standard, however I have significant reservations, as outlined above.
